# Antiulcer Agents: From Plant Extracts to Phytochemicals in Healing Promotion

**DOI:** 10.3390/molecules23071751

**Published:** 2018-07-17

**Authors:** Mehdi Sharifi-Rad, Patrick Valere Tsouh Fokou, Farukh Sharopov, Miquel Martorell, Adedayo Oluwaseun Ademiluyi, Jovana Rajkovic, Bahare Salehi, Natália Martins, Marcello Iriti, Javad Sharifi-Rad

**Affiliations:** 1 Department of Medical Parasitology, Zabol University of Medical Sciences, Zabol 61663335, Iran; mehdi_sharifirad@yahoo.com; 2 Department of Biochemistry, Faculty of Science, University of Yaounde I, Yaounde Po.Box 812, Cameroon; tsouh80@yahoo.fr; 3 Department of Pharmaceutical Technology, Avicenna Tajik State Medical University, Rudaki 139, Dushanbe 734003, Tajikistan; shfarukh@mail.ru; 4 Nutrition and Dietetics Department, School of Pharmacy, University of Concepción, Concepción 4070386, VIII–Bio Bio Region, Chile; mmartorell@udec.cl; 5 Functional Foods, Nutraceuticals and Phytomedicine Unit, Department of Biochemistry, Federal University of Technology, Akure 340001, Nigeria; ademiluyidayo@yahoo.co.uk; 6 Institute of Pharmacology, Clinical Pharmacology and Toxicology, Medical Faculty, University of Belgrade, Belgrade 11129, Serbia; jolarajkovic@yahoo.com; 7 Medical Ethics and Law Research Center, Shahid Beheshti University of Medical Sciences, Tehran 88777539, Iran; 8 Student Research Committee, Shahid Beheshti University of Medical Sciences, Tehran 22439789, Iran; 9 Faculty of Medicine, University of Porto, Alameda Prof. Hernâni Monteiro, Porto 4200-319, Portugal; 10 Institute for Research and Innovation in Health (i3S), University of Porto–Portugal, Porto 4200-135, Portugal; 11 Department of Agricultural and Environmental Sciences, Milan State University, via G. Celoria 2, Milan 20133, Italy; 12 Phytochemistry Research Center, Shahid Beheshti University of Medical Sciences, Tehran 11369, Iran; 13 Department of Chemistry, Richardson College for the Environmental Science Complex, The University of Winnipeg, Winnipeg, MB R3B 2G3, Canada

**Keywords:** peptic ulcer, *Helicobacter pylori*, gastric cancer, bioactive phytochemicals, herbal remedies, traditional healing systems

## Abstract

In this narrative review, we have comprehensively reviewed the plant sources used as antiulcer agents. From traditional uses as herbal remedies, we have moved on to preclinical evidence, critically discussing the in vitro and in vivo studies focusing on plant extracts and even isolated phytochemicals with antiulcerogenic potential. A particular emphasis was also paid to *Helicobacter pylori* activity, with emphasis on involved mechanisms of action. Lastly, the issue of safety profile of these plant products has also been addressed.

## 1. Introduction

Peptic ulcers (PU) are sores or lesions in the gastrointestinal mucosa extending throughout the muscularis mucosae, typically characterized by different stages of necrosis, neutrophil infiltration, blood flow reduction, increased oxidative stress and inflammation [1]. PU manifest as a non-fatal disease, majorly represented by periodic symptoms of epigastric pain, which are often relieved by food or alkali, besides to trigger much discomfort to patients, disrupting their daily routines and also causing mental agony [2]. 

The disease is mostly categorized based on its anatomical origins, such as gastric (found along the lesser curvature of the stomach) and duodenal (occurring in the duodenal bulb—the most exposed area to gastric acid) ulcers [3]. Studies have shown that peptic ulcer disease (PUD) occurs because of an imbalance between aggressive injurious (e.g., pepsin, HCl) and defensive mucosa-protective factors (e.g., prostaglandins, mucus and bicarbonate barrier and adequate blood flow) [4]. All ulcers of the upper gastrointestinal tract were originally thought to be caused by the aggressive action of pepsin and gastric acid on mucosa. However, the denomination “peptic ulcer” has lately pointed to *Helicobacter pylori* infection, where the chronic use of non-steroidal anti-inflammatory drugs (NSAIDs) and acetylsalicylic acid (ASA) are some of the disease-causing factors.

Thus, based on the latest advances on this field and stress the fact that PUD is an important cause of morbidity and health care costs, the present report aims to provide a general overview on peptic ulcers, namely considering their epidemiology, main symptoms and clinical features, pathogenesis, where a particular emphasis will be given to *H. pylori* infection, pharmacological agents used in an effective management and also pointing out the latest challenges and opportunities of using plant phytochemicals as upcoming antiulcerogenic agents. Lastly, a special emphasis was given on plant products safety and security, in order to trigger the interest in deepening skills on this matter and to ensure an effective managing competence for health-related systems. 

## 2. Epidemiology of Peptic Ulcer

Epidemiological studies have established that the prevalence of PU is a reflection of *H. pylori* infection prevalence, increasing with NSAIDs and ASA use, as also with ageing population. PUD had a tremendous effect on morbidity and mortality until the last decades of 20th century, when a significant decline in its incidence was observed [5]. This dramatic shift in the prevalence pattern of the disease was correlated to changes in environmental factors, such as modernization. Moreover, it was hypothesized that through improving hygiene and overall health quality in developed countries may have resulted in reduced rates of childhood infections and *H. pylori* spread [6]. Also, two more important findings were stated as influencing the decrease in PUD rates: the discovery of effective and potent acid suppressants and *H. pylori* infection treatment and prevention. However, by the turn of the century, the increased use of NSAIDs has resulted in a decline in duodenal ulcers (*H. pylori*-associated infection) and increase in gastric ulcers (NSAIDs-caused ulcers) [7]. Nevertheless, PU still remain common worldwide, especially in developing countries, where *H pylori* infection is highly prevalent [8]. Studies have revealed that a high percentage of children are infected with *H. pylori* before the age of 10 and the prevalence peaks at more than 80% before the age of 50 in the developing countries [9]. However, serologic evidence of *H. pylori* infection is uncommon before age 10 in developed countries, such as US but increases to 10% between 18 and 30 years and to 50% in ages above 60 years [9]. Indeed, the lifetime risk for developing a PU is estimated to be approximately 10% [10]. Anyway, only a minority of cases with *H. pylori* infection will lead to an ulcer, while a larger proportion of people will get non-specific discomfort, abdominal pain or gastritis.

## 3. Symptoms and Clinical Features of Peptic Ulcer

Most patients with PU present abdominal discomfort, pain or nausea, with epigastric pain being the most common PU symptom (gastric and duodenal ulcers). This is characterized by a gnawing or burning sensation occurring after meals. Usually, duodenal ulcer pains can be relieved with food or antacids [11], while gastric ulcer pain is often aggravated by meals. Furthermore, duodenal ulcer is associated with nightly pain occurring in about 50–80% of sufferers as opposed to about 30–40% in gastric ulcer patients. Pains radiating to the back may suggest that an ulcer has penetrated posteriorly, or the pain may be from pancreatic origin. Other possible manifestation of the disease includes dyspepsia, such as belching, bloating, distention, intolerance to fatty foods, weight loss, or poor appetite [11]; heartburn, chest pain/discomfort, hematemesis and even anemia [12]. Patients may often be asymptomatic with only 20–25% of them having suggestive symptoms of peptic ulceration, found to have peptic ulcer after investigation.

## 4. Pathogenesis

The pathogenesis of PU has been found to be complex and multifactorial. However, one common feature in its pathogenesis is the imbalance between aggressive luminal factors (gastric acid and pepsin) and defensive mucosal barrier function [5]. Critical to trigger gastric ulceration are the contribution of several host and environmental factors, which increase gastric acid secretion, and/or weaken mucosal barriers. Emotional stress, smoking, nutritional deficiencies, excessive alcohol consumption and prolonged ingestion of NSAIDs are relevant etiological environmental factors contributing to PU development [5,13,14]. Furthermore, *H. pylori* infection has been known to play central role in the development of chronic gastritis, gastric ulcers, duodenal ulcers and gastric cancer [15]. *H. pylori* triggers chronic gastritis by gastric epithelium infiltration and underlying lamina propria by immune cells, such as neutrophils, macrophages, lymphocytes and mast cells. Also, *H. pylori* produces some toxic biomolecules to the epithelial cells, such as ammonia produced to regulate stomach pH, proteases and vacuolating cytotoxin A, which damages epithelial cells and could cause apoptosis and certain phospholipases [16]. Else, *H. pylori*-derived biomolecules, such as lipopolysaccharide (LPS) and cysteine-rich proteins (Hcp), particularly HcpA (hp0211), have been shown to trigger immune response and contribute to inflammation [17,18]. Therefore, chronic gastritis (inflammation of the stomach lining) can be considered as resulting from stomach bacterial colonization at sites of infection. 

Studies have established an association between chronic NSAIDs use and incidence of PU in the developed world. This was consequent upon the discovery and treatment of *H. pylori* infection. In fact, it has been reported that chronic NSAIDs use could damage gastric and duodenal mucosa via several mechanisms, such as drugs-induced topical irritation of the epithelium, impairment of the barrier properties of mucosal membrane, suppression of prostaglandins synthesis in the gastric area, reduction of blood flow to the gastric mucosal and interference with superficial injury repair [19]. Anyway, the presence of acid in the stomach lumen also contributes to the pathogenesis of NSAID-induced ulcers and bleeding, through impairing hemostasis and inhibiting growth factors that are important in mucosal defense and repair [19].

## 5. Pharmacological Agents Effective in Disease Management

Pharmacological management of PU continues to evolve despite the availability of diverse types of new therapeutic agents, with the aim of centering treatment on pain relieving, ulcer healing and in ulcer recurrence delay. However, many of the pharmacological agents available for treating PU are targeted at either counteracting the aggressive factors or stimulating the mucosal defense. Such drugs (Table 1), targeted to inhibit/neutralize gastric acid secretion, includes antacids, histamine H2-receptor antagonists, proton pump inhibitors, anticholinergics and prostaglandins [20].

## 6. Plant Products and Phytochemicals as Antiulcerogenic and Gastroprotective Agents

Since ancient times, plants and plant derived-products have been used in folklores around the world for the treatment of several ailments and diseases. Nowadays, herbal medicine is becoming a viable alternative treatment over the commercially available synthetic drugs on PU management/treatment. This is premised on its lower cost, perceived effectiveness, availability as well as little or no adverse effects. A number of these herbal remedies have demonstrated gastroprotective properties [2,27,28,29] and have been used in the treatment of PU, digestive disorders and other related ailments for several centuries. 

### 6.1. Materials and Methods

An extensive search of existing literature was performed and carefully collected from all scientific journals (original research, reviews, short communications), books and reports from worldwide accepted databases (Scopus, ScienceDirect, PubMed, Web of Science, Medline, Springer and Google Scholar). The following keywords were searched, “*Helicobacter pylori*,” “infection”, “peptic ulcer”, “bioactive molecules”, “phytochemicals”, “plant species”, “antiulcerogenic”, “gastroprotective”. In addition, the biography of all selected articles was accurately handled in seeking for additional relevant articles.

### 6.2. Plant Extracts with Antiulcerogenic Activity

This section reports the plant extracts with antiulcerogenic activity that are also briefly summarized in Table 2.

Among the studied plant extracts, those belonging to the Asteraceae, followed by the Combretaceae and Fabaceae families, were the most frequently studied and were reported to have promising wound healing, antioxidant, anti-inflammatory, cytoprotective, gastric secretion inhibition, mucus production improvement, HSP70 up-regulation, Bax protein down-regulation, anti-secretory and anti-*H. pylori* effects. Below, are briefly described all the studied plant extracts with antiulcerogenic activity, its corresponding modes of action and studied models.

#### 6.2.1. *Centella asiatica*

*Centella asiatica* (L.) Urban is a very important medicinal herb used in the Orient. In China, it is called gotu kola and is one of the reported “miracle elixirs of life,” known of over two thousand years ago. *C. asiatica* leaf extract (50 or 250 mg/kg) evidenced a pronounced gastroprotective activity against indomethacin-induced ulcer in rats [30], that could be attributed to its ability to inhibit lipid peroxidation and to stimulate gastric mucus secretion in the rat gastric mucosa [30]. Several studies confirmed the ulcer-protective effects of this plant in various animal models [31].

#### 6.2.2. *Baccharis dracunculifolia*

*Baccharis dracunculifolia* DC is a native plant species from Brazil and represents the main source of southeastern Brazilian propolis, commonly known as green propolis. The essential oil from its leaves, rich in non-oxygenated and oxygenated terpenes, showed antiulcer activity in rats (at doses 50, 250 and 500 mg/kg), lowering the lesion index, total lesion area and the percentage of lesion, with a consequent reduction of gastric juice volume and total acidity [33]. In another study, the chemical composition of *B. dracunculifolia* leaves, in terms of phenolic compounds, was accessed and investigated its bioactive potential in rats with trinitrobenzenosulfonic acid (TNBS)-induced ulcerative colitis [32]. The oral dose of 5 and 50 mg/kg plant extract attenuated the damage induced by TNBS and improved the colonic oxidative status, mainly through reducing myeloperoxidase (MPO) activity and empowering endogenous antioxidant defenses, such as glutathione (GSH) levels and inhibited lipid peroxidation [32].

#### 6.2.3. *Baccharis trimera*

*Baccharis trimera* (Less.) DC, popularly known as “carqueja” in Brazil, is widely recognized as a treatment for gastrointestinal, hepatic and kidney disorders and diabetes. The anti-secretory and antiulcer activity of *B. trimera* aqueous extract (1000–2000 mg/kg) was already reported in stress-induced ulcer model and pylorus ligature model [34] and even in a model of hydrochloric-induced acute gastric ulcers, where a lower dose (100–400 mg/kg) displayed antiulcer activity too [35]. In a more recent research performed, using a lower dose and a more concentrated *B. trimera* extract (90%), it was shown that the oral administration of 30 mg/kg significantly reduced the lesion area and the macroscopic appearance of acute and chronic ulcer models [36]. Also, in a study performed using high doses (1000–2000 mg/kg), the authors proposed that *B. trimera* inhibits gastric acid secretion by acting on the cholinergic regulatory pathway. However, Lívero et al. [36] proposed that the effects of *B. trimera* extract in preventing or reversing ethanol- and acetic acid-induced ulcers may be attributable to the inhibition of free radical generation and subsequent prevention of lipid peroxidation and that flavonoids and caffeoylquinic acids detected in the studied extract play an important and contributive role on these effects.

#### 6.2.4. *Hieracium gymnocephalum*

Some species from *Hieracium* L. genus (Compositae) are used as anti-inflammatories and diuretics in traditional European Medicine. A study performed with a chloroform extract of *H. gymnocephalum* blooms at 25, 50, 100 and 200 mg/kg, in Wistar rats with indomethacin-induced acute ulceration, suggested that the extract has anti-inflammatory and gastroprotective effects, though poor antioxidant activity [37]. Based on these findings, the authors proposed that triterpene alcohols present in the *H. gymnocephalum* chloroform extract were the main contributors to observed anti-inflammatory and gastroprotective effects.

#### 6.2.5. *Tanacetum larvatum*

*Tanacetum larvatum* (Griseb. ex Pant.) Kanitz is an endemic perennial herb distributed on rocky places in Serbia, Montenegro and Albania. The chloroform extract from the aerial blooming parts of *T. larvatum* showed a dose-dependent anti-inflammatory activity, with significantly less gastric lesions at 200 mg/kg dose in Wistar rats ulcered with indomethacin [38]. The authors proposed that these effects might be mainly due to DNA binding inhibition of the transcription factor NF-κB by distinct components of the plant extract. Moreover, it has been discussed that this effect may also be attributed to its ability to restore the reduction of sulfhydryl groups within the gastric mucosa [114] and to enhance the mucosal PGE2 levels [115].

#### 6.2.6. *Vernonia condensata*

The leaves from *Vernonia condensata* Baker are traditionally used in folk medicine, being this plant widely known in Brazil. The gastroprotective and gastric healing properties of a crude ethanolic extract from leaves of this plant (3, 30, 300 mg/kg) were evaluated in Wistar rats and mice ulcered using ethanol and indomethacin [39]. The results of this study showed that higher doses (30 and 300 mg/kg) reduced lesion size in indomethacin-induced ulcer model [39], similarly to a previous report on a polar fraction of *V. condensata* (200 mg/kg) in the same model [40]. Therefore, *V. condensata* extract presents an antiulcer effect, mediated through inhibition of gastric secretion via cholinergic and gastrinergic pathways and produces a cytoprotective effect by increasing antioxidant activity and mucin content and also inhibits neutrophil migration [39]. 

#### 6.2.7. *Solidago chilensis*

*Solidago chilensis* Meyen—popularly known as “vara dorada”—is a native plant from South America, widely used in folk medicine as an anti-inflammatory, diuretic and for gastrointestinal disorders. The gastroprotective effect of *S. chilensis* methanol leaf extract (100 and 300 mg/kg) was evaluated in gastric ulcer models of Swiss mice and in L929 cells [41]. The results of this study showed that leaf extract promotes gastroprotection and exerts gastric healing benefits through diversified and complementary modes of action because of the presence of flavonoids (especially quercitrin and afzelin), which are related to its antioxidant and anti-secretory properties in parallel to its beneficial effect on mucus production [41]. In another study performed, using the aqueous extract from *S. chilensis* inflorescences (125, 250, 400, 800, 1200 and 2000 mg/kg) in albino mice subjected to ethanol-induced gastric ulcer model, a significant antiulcer activity was also demonstrated [42].

#### 6.2.8. *Cordia dichotoma*

To *Cordia dichotoma* G. Forst, commonly known as “Bhokar” in Marathi, are attributed many medicinal properties in Ayurveda Medicine. The methanol fraction of the crude methanol extract of *C. dichotoma* bark (500 mg/kg) showed a protective role against acid acetic-induced ulcerative colitis in Swiss mice trough anti-inflammatory and antioxidant mechanisms [43].

#### 6.2.9. *Moringa oleifera*

*Moringa oleifera* L.—also known as horseradish tree and rumstick tree—is a perennial plant indigenous to Northwest India, Pakistan, Bangladesh and Afghanistan and commonly used for medicinal and nutritional purposes. The ethanolic root-bark extracts of *M. oleifera* (150, 350 and 500 mg/kg) were tested as antiulcer agent in albino Wistar rats with ethanol-induced and pylorus ligation-induced gastric ulceration models, being stated prominent antiulcer, anti-secretory and cytoprotective abilities [46]. Moreover, the hydroalcoholic extract (50, 100 and 200 mg/kg) and its chloroform fraction (100 and 200 mg/kg) from *M. oleifera* seeds showed therapeutic effects in Wistar rats with acetic acid-induced colitis, even causing a significant reduction of ulcer severity, area and index as well as on mucosal inflammation severity and extent, crypt damage, invasion involvement, total colitis index and myeloperoxidase activity [47]. On the other hand, the aqueous leaf extract (50–500 mg/kg) of this plant was also able to prevent gastric ulceration in Holtzman strain albino rats ulcered using aspirin through potentiation of serotonin release [45].

#### 6.2.10. *Capparis zeylanica*

*Capparis zeylanica* L. is widely recognized in traditional Ayurvedic Medicine. The methanolic extract from its leaves (200 mg/kg) exhibited a stomach-protective effect against ethanol necrotic damage in a study performed using three different models (ethanol, aspirin and indomethacin) of induced ulcers in albino rats [44]. The authors suggested that ulcer protection might be attributed to the phytochemicals present in *C. zeylanica* leaves, among them flavonoids, tannins and saponins.

#### 6.2.11. *Salvadora indica*

*Salvadora indica* Royle—commonly known as “jhak” in Hindi—is traditionally used as an important key ingredient in tooth care products by Arabian people. A study performed with the ethanolic extract of *S. indica* leaves (150, 300 and 600 mg/kg), using pylorus ligation, ethanol and cysteamine induced ulcer models in albino rats, showed a dose-dependent cytoprotective effect against ethanol-induced cellular damage in gastric mucosa [48]. Flavonoids, tannins and triterpenoids, present in the extract, seems to be responsible for the antiulcer effect.

#### 6.2.12. *Maytenus robusta*

*Maytenus robusta* Reissek is used in Brazilian folk medicine to treat gastric ulcers and its gastroprotective properties have been demonstrated in acute gastric ulcer models [27,28]. The healing effect of *M. robusta* in chronic gastric ulcer has been attributed to its ability to empowering protective factors of the gastric mucosa, such as mucosa layer regeneration, antioxidant defenses and cell proliferation [49]. In fact, the gastroprotective effects of *M. robusta* hydroalcoholic extract were demonstrated in acute gastric ulcer models, where particularly leaves (50, 250 and 500 mg/kg) [27] and 15-dioxo-21alpha-hydroxy friedelane (150 mg/kg) [28] also displayed anti-secretory effects. The efficacy of hydroalcoholic extract from *M. robusta* aerial parts was evaluated in Wistar rats with acetic acid-induced chronic ulcer model and in L929 cells, to determine its effect on cell proliferation, free radicals scavenging and inflammatory and oxidative damages [49]. The results of this study showed that the oral administration of extract (10 mg/kg) reduced the gastric ulcer area, increasing gastric mucin content and reducing oxidative stress and inflammatory parameters at the ulcer site. An in vitro study demonstrated that the hydroalcoholic extract (1–1000 µg/mL) promoted cytoprotection against H_2_O_2_ and free radical scavenging [49]. In another study, 3,12-dioxofriedelane and 11-hydroxylup-20(29)-en-3-one and mayteine and 3,7-dioxofriedelane were isolated from the *n*-hexane and dichloromethane fraction of *M. robusta* root barks, respectively; their gastroprotective activity was evaluated in mice with ethanol and NSAIDs-induced ulcer models [50]. The results showed that the crude extract (50, 250, 500 mg/kg) and all fractions (250 mg/kg) reduced the lesion index, total lesion area and percentage of lesions, while all the isolated compounds also presented significant pharmacological effects at 30 mg/kg [50]. Also, studies have revealed that *M. robusta* is rich in pentacyclic triterpenes [28,116,117] and, consequently, the gastric health promoting effects of *M. robusta* have been ascribed to the ability of pentacyclic triterpenes to stimulate mucus synthesis and prostaglandin secretion, thus strengthening gastric mucosa defense factors [28,118]. However, other phytochemicals such as steroids and flavonoids could also have contributed to the antiulcer properties of this plant [119].

#### 6.2.13. *Mukia maderaspatana*

*Mukia maderaspatana* (L.) M. Roem leaves are commonly used in Siddha and Ayurvedic Medicine. The ethanolic extract from *M. maderaspatana* was able to reduce gastric mucosal lesions, malondialdehyde (MDA) and TNF-α levels, while increased gastric juice mucin content and gastric mucosal catalase (CAT), nitric oxide and PGE2 levels in rats with indomethacin-induced gastric ulcer [52]. These biological effects suggest a gastric protection conferred by its antioxidant, anti-inflammatory and mucin-enhancing properties. 

#### 6.2.14. *Momordica cymbalaria*

*Momordica cymbalaria* Hook f. is well-known as “Athalakkai” in Tamil and it is available in various parts of India, where *M. cymbalaria* fruits decoction are used in traditional medicine as a treatment for gastric ulcers. Wistar rats with ethanol-induced ulcer were treated with 500 mg/kg of the aqueous extract from *M. cymbalaria* fruits and showed a decrease in total acidity, ulcer index and gastric lesions, while histopathological parameters improved [51]. Further, it was reported that the aqueous extract is rich in the flavanol quercetin, which was earlier reported for its antiulcer effects [120] and other flavonoids, cucurbitacins, momordicine and glycosides, which possess antioxidant properties and stimulate prostaglandin secretion [51,121].

#### 6.2.15. *Cibotium barometz*

*Cibotium barometz* (L.) J. Smith is a tropical and sub-tropical plant, also known as “golden hair dog fern,” used as medicinal plant in Malaysian Peninsula and parts of China. The gastroprotective effect of the ethanolic extract from its leaves [53] and hairs [54] was evaluated against ethanol-induced stomach ulcers in Sprague Dawley rats at 62.5, 125, 250 and 500 mg/kg. The *C. barometz* leaves presented a dose-dependent antiulcer effect, increased antioxidant enzymes activity, such as superoxide dismutase (SOD), CAT and glutathione peroxidase (GPx) and decreased lipid peroxidation [53]. On the other hand, *C. barometz* hairs presented a dose-dependent antiulcer effect too, free radical scavenging activities, increased pH, gastric mucus glycoprotein and antioxidant enzymes activity (SOD, CAT and GPx) and reduced MDA levels [54]. Moreover, the effects of both *C. barometz* leaves and hairs were proposed as being related to HSP70 up-regulation and Bax protein down-regulation [53,54]. 

#### 6.2.16. *Cyperus rotundus*

*Cyperus rotundus* L. a widely used plant against gastric ailments in traditional Indian Medicine, especially in Ayurveda. The oral administration of methanolic extract from *C. rotundus* rhizomes (250 and 500 mg/kg) inhibited aspirin-induced ulceration in Wistar rats in a dose-dependent manner, being even comparable with standard drug ranitidine (50 mg/kg) [55]. Moreover, the same study showed that this extract inhibited oxidative damage in gastric mucosa through increasing antioxidant enzymes activity (SOD, GSH and GPx) [55]. Also, in another study, this plant exhibited anti-inflammatory and antiulcer activities at doses of 300 and 500 mg/kg [56].

#### 6.2.17. *Caesalpinia sappan*

*Caesalpinia sappan* L.—widely known as pattanga—has been used in the Ayurveda system of medicine for a long time. The hydroalcoholic extract from *C. sappan* heartwood is rich in polyphenols, including flavonoids such as brazilin, brazilein, hydroxybrazilin and peonidin-3-galactoside which display an interesting antioxidant activity [57]. The effectiveness of this extract on ulcer protection was also assayed, being of 92%, 86% and 64% against ethanol, indomethacin and pylorus ligation induced ulcers in Wistar rats, respectively. Moreover, the plant extract exhibited cytoprotective effects with 76.82% reduction against indomethacin-induced cytotoxicity at the concentration of 25 µg/mL and showed 63.91% inhibition in H^+^/K^+^-ATPase (proton pump) inhibitory assay at 500 µg/mL. Indeed, the authors proposed that the gastroprotective activity of *C. sappan* heartwood is possibly mediated by cytoprotective and antioxidant mechanisms involving an increase in PGE2 synthesis and a decrease in myeloperoxidase (MPO) levels, conferred by antioxidant phytoconstituents present in the plant [57].

#### 6.2.18. *Archidendron jiringa*

*Archidendron jiringa* (Jack) I.C. Nielsen is native from Southeast Asia and belongs to the Fabaceae family. *A. jiringa* ethanol extract (250 and 500 mg/kg) was protective against ethanol-induced gastric mucosal ulcers in Sprague Dawley rats, increasing adherent mucus and inhibiting oxygen-derived free radicals generation (through increasing SOD activity and decreasing MDA levels) [58].

#### 6.2.19. *Alhagi maurorum*

*Alhagi maurorum* Medik, namely its aerial parts, have been traditionally used and studied for multiple purposes. Shaker et al. [59] investigated the antiulcerogenic and anti-inflammatory effects of an ethanolic extract from this plant in aspirin-induced ulcer model on Sprague–Dawley rats. The plant extract (100 mg/kg) protected liver enzymes, improved oxidation status (MDA and GSH) and fucosidase tumor markers though no ulcer pattern was evident in the histopathology [59].

#### 6.2.20. *Cassia sieberiana*

*Cassia sieberiana* DC. is a savannah tree whose roots have been traditionally used in the management of various stomach disorders, including gastric ulcer, stomach pains and indigestion [60]. In fact, it was shown that the root bark extract from this plant exhibited a prominent gastric cytoprotective property, which was associated with its antioxidant properties as well as the stimulatory effect on gastric mucosal prostaglandin E2 (PGE2) and prostaglandin I2 (PGI2) levels with a resultant decrease in serum phospholipase A2 (sPLA2) activity [61]. 

#### 6.2.21. *Tamarindus indica*

*Tamarindus indica* L. is a plant tree indigenous from the tropical Africa and America but also cultivated in several regions, such as China, India, Philippines and Spain. The methanolic extract of the seed coat of this plant (100 mg/kg and 200 mg/kg) has been evaluated for determining their antiulcer potential on ibuprofen, alcohol and pyloric ligation-induced gastric lesions using albino Wistar rats [63]. The results of this study showed that the methanolic extract reduced total gastric juice volume and free and total gastric secretion acidity in pylorus ligation-induced ulcer model, while reduced ulcer index (comparable with ranitidine, 50 mg/kg, as control) [63].

#### 6.2.22. *Parkia speciosa*

*Parkia speciosa* Hassk—also known as stink bean—is popular in Southeastern Asia, besides having medicinal properties. A study performed to establish the antioxidant and antiulcer activity of *P. speciosa* ethanol leaf extract (50, 100, 200 and 400 mg/kg) against ethanol-induced gastric ulcers in rats concluded that the extract is effective [62]. The results of this study showed that the extract may act by enhancing the gastric mucosal defense and/or by inhibiting leukotriene synthesis. Treatment with the extract led to heat-shock protein 70 (HSP70) upregulation and pro-apoptotic protein Bax downregulation. Moreover, in gastric mucosa, the extract increased antioxidant enzymes (GSH and SOD) and decrease MDA levels [62].

#### 6.2.23. *Morinda citrifolia*

*Morinda citrifolia* L.—also known as ′noni′—is a plant native from Southeast Asia and is commonly used in popular medicine in Brazil. The health benefits of fruit aqueous extract (0.63 to 2.50 g/kg) and its isolated compound, scopoletin, were evaluated in models of gastro-esophageal inflammation in rats (acid reflux esophagitis, acute gastritis induced by ethanol and serotonin and chronic gastric ulcer induced by acetic acid) [64]. The studied extract was able to inhibit acid reflux esophagitis, reduced gastric lesions formation induced by alcohol and serotonin and accelerated gastric ulcers healing induced by acetic acid. Moreover, isolated scopoletin also produced similar effects, though its anti-secretory and prokinetic activities included an inhibitory activity on serotonin, free radicals and cytokine-mediated inflammation [64]. 

#### 6.2.24. *Kigelia africana*

*Kigelia africana* (Lam.) Benth is a native plant from the South-western part of Nigeria, where hot-infusion preparations of its leaves are popularly used to treat stomach ulcers. Ethanolic leaf extract from this plant presented antiulcerogenic potential against aspirin-induced ulcer, while aqueous leaf extract against ethanol-induced ulcer in Wistar albino rats [65]. The antiulcer effect of *K. africana* can be attributed to the presence of antioxidant flavonoids (e.g., rutin, quercetin and kaempferol). 

#### 6.2.25. *Cordia verbenacea*

*Cordia verbenacea* DC is a shrub medicinal plant popularly used in Brazil, where leaf infusion or decoction are widely used for anti-inflammatory, analgesic, antiulcer and anti-rheumatic purposes. The antiulcer activity of *C. verbenacea* ethanol leaf extract (125, 250, 500 or 1000 mg/kg) was evaluated using ethanol/HCl, ethanol and piroxicam-induced gastric lesion models, pylorus-ligated assay [66]. In this study, a potent antiulcer activity of the leaf extract (125 mg/kg) in ethanol/HCl and absolute ethanol-induced gastric lesions was reached [66]. Moreover, the authors of this study associated the gastroprotective effect to an improvement of antioxidant mechanisms and to a light involvement on nitric oxide in stomach mucosa [66]. 

#### 6.2.26. *Barleria lupulina*

*Barleria lupulina* Lindl is a plant widely distributed in Indian mountains and is used in the folk culture of India, Thailand and Pakistan. The gastroprotective effect of the methanol extract from *B. lupulina* aerial parts was tested in Wistar rats using various models of ulcers, such as drug induced ulcers, restraint ulcers, duodenal ulcers and pylorus ligated ulcers [68]. This study showed that the methanolic extract tested dose of 200 mg/kg has a protective effect against experimental gastric and duodenal ulcers, reduced gastric juice volume, total acidity and ulcer index in pylorus ligated rats [68]. Moreover, the methanol extract from *B. lupulina* aerial parts (300 mg/kg) exhibited anti-inflammatory activity without displaying ulcerogenic activity in rats [67].

#### 6.2.27. *Eremomastax speciosa*

*Eremomastax speciosa* (Hochst.) Cufod. is a widely distributed plant in tropical Africa and used in Cameroonian ethnomedicine, where it is commonly referred to as “blood plant” due to its reputed use in the treatment of anemia. In 1996, it was reported that the water extract from *E. speciosa* leaves (190 mg/kg) inhibited the formation of HCl/ethanol-inflicted and pylorus ligation gastric lesions in rats [69]. In 2013, the protective effect of the whole methanol plant extract (100–200 mg/kg) was demonstrate, using various experimental ulcer models, including HCl/ethanol, absolute ethanol, cold/restraint stress and indomethacin. The observed benefits seems to be due to its ability to reduce acid secretion and to enhance mucosal defense and antioxidant status [70]. The authors of this last experiment, performed another study for evaluating the effect of different extracts from *E. speciosa* aerial parts (200–400 mg/kg) against carbachol- and histamine-induced hypersecretion, associated with the pylorus ligation technique, in Wistar rats [71]. As the main findings, it was shown that the water and MeOH-CH_2_Cl_2_ extracts and even CH_2_Cl_2_ fraction of *E. speciosa* protected the gastric mucosa and inhibited gastric acid secretion [71]. Interestingly, the aqueous extract seemed to offer cytoprotection through reinforcement of gastric mucous layer or through exerting similar effects to the endogenous prostaglandins and anti-secretory effect through cholinergic and histaminergic pathways modulation [71].

#### 6.2.28. *Hyptis suaveolens*

*Hyptis suaveolens* (L.) Poit. is originally a native plant from tropical America but now considered a worldwide weed and used in folk medicine in several parts of the world. The ethanolic extract and hexane fraction from the aerial parts of this plant (62.5, 125, 250 and 500 mg/kg) were evaluated in models of acute gastric ulcer (ethanol-induced, hypothermic restraint-stress, NSAID-induced, pyloric ligation-induced) and mucosal lesions, using *Rattus novergicus* rats, Wistar strain and *Mus musculus* [73]. The results of this study showed that these plant extracts were able to reduce gastric lesions induced by all ulcerogenic agents tested, which supports the use of this plant as a gastroprotective agent [73]. Suaveolol, an abietane diterpene, was identified as the main active gastroprotective agent in *H. suaveolens,* where its mode of action involves nitric oxide, prostaglandins and sulfhydryl groups [74].

#### 6.2.29. *Tectona grandis*

Verbascoside, a phenolic glycoside isolated from *Tectona grandis* L. evidenced a prominent ability to mediate gastric protection in experimental animals via inhibiting proton pump (H^+^/K^+^-ATPase) activity with a corresponding decrease in plasma gastrin level [75], similarly to the *Solanum nigrum* L. fruit extract [76]. 

#### 6.2.30. *Calamintha officinalis*

*Calamintha officinalis* Moench, widely known as “Mentuccia” in central Italy, is an aromatic plant used for preservative and medicinal purposes since ancient times. The methanol extract obtained from its leaves (12.9% *w*/*w*) displayed an important antiulcerogenic activity in rats ulcered with ethanol, being the observed effect similar to sucralfate, a drug that has a protective action on gastric lesion induced by ethanol [72]. The authors suggested that the gastroprotective effects may depend on the synergistic action of all compounds present in *C. officinalis* extract, namely polyphenols, catechin, tannins and terpenes, even being able to remove damaging agents from the gastric mucosa conferred by its prominent antioxidant activity [72].

#### 6.2.31. *Myristica malabarica*

*Myristica malabarica* Lam., mainly its fruit rind, is used as an exotic spice in Indian cuisine. Malabaricones B and C are active constituents present in methanol extract [79], which at a dose of 40 mg/kg could heal indomethacin-induced stomach ulceration in mice, through reducing the ulcer index [80]. The extract exhibited more potent antiulcer effect than the individual malabaricones [79]. The gastroprotective effect of *M. malabarica* extract involves vascular endothelial growth factor (VEGF) enhancing and endostatin levels reduction, relevant mechanisms in angiogenesis and prostaglandin synthesis [77]. Moreover, malabaricone B regulates arginase/nitric oxide synthesis, through modulation of anti/pro-inflammatory cytokine ratio, that contributes to the healing action against indomethacin-induced gastric ulceration in mice [78].

#### 6.2.32. *Talinum portulacifolium*

*Talinum portulacifolium* (Forssk.) Asch. ex Schweinf, namely its leaves, have been used for a long time by Indian people against diabetes, fevers and ulcer. Whole plant ethanolic extract (800 mg/kg) showed antiulcer activity on albino rats in three models of induced gastric ulcers: ethanol, pylorus ligated aspirin and histamine [81]. This extract enhanced gastric volume, pH of gastric juice, total acidity, free acidity and ulcer index.

#### 6.2.33. *Cratoxylum arborescens*

*Cratoxylum arborescens* (Vahl) Blume is widely distributed in Sabah and Sarawak (Malaysia) and its bark, roots and leaves are used in traditional medicine. Different xanthones, α-mangostin and β-mangostin have been isolated and identified from *C. arborescens* and have been studied as antiulcer protectors [84] and anti-*H. pylori* agents [83,84]. A study performed with α-mangostin showed that this compound (10 and 30 mg/kg) was able to protect gastric mucosa against ethanol-induced injury on rats, through exerting antioxidant protection, interfering with nitric oxide release, cyclooxygenases (COXs) inhibition and anti-*H. pylori* activity [84]. In another study, β-mangostin (5, 10 and 20 mg/kg) exhibited gastroprotective activity due to mucus production, anti-secretory, antioxidant, antiapoptotic and anti-*H. pylori* activity [83].

#### 6.2.34. *Mammea americana*

*Mammea americana* L.—commonly known as mammee—is an evergreen tree of the Calophyllaceae family, the fruit of which is edible. In folk medicine, this plant is used against stomachache. Three different bark/latex extracts from this plant (ethanol, methanol and dichloromethane) has been evaluated, in Swiss mice, for their ability to confer gastric mucosa protection against necrotizing agents, hypothermic restrain stress, NSAIDs and pylorus ligation [82]. In all gastric ulcer models tested, the authors did not find any significant effect conferred by methanol extract; nevertheless, ethanol and dichloromethane extracts showed excellent anti-secretory and/or gastroprotective effects in all gastric ulcer-induced models used. These results suggest that the apolar fraction obtained from *M. americana* bark/latex extracts retain antiulcerogenic compounds [82].

#### 6.2.35. *Terminalia catappa*

*Terminalia catappa* L. is a plant widely distributed in tropical and subtropical regions and listed in Caribbean pharmacopeia as a medicinal plant to treat gastritis. The aqueous extract from leaves (25 mg/kg) showed preventive and curative effects on acute and chronic induced gastric ulcers on rats (absolute ethanol and ischemia-reperfusion injury) and an important inhibitory profile against *H. pylori* [88]. The authors suggested that the mechanisms involved on *T. catappa* gastroprotective effects are related to nitric oxide pathway, increasing endogenous prostaglandins levels and mucus production and inhibiting MMP-9 and MMP-2 activities [88].

#### 6.2.36. *Terminalia coriacea*

*Terminalia coriacea* (Roxb.) Wight & Arn. is a plant found in India, usually used as cattle feed and in Ayurveda and Siddha traditional medicine to heal ulcers. A leaf methanol extract (125, 250 and 500 mg/kg) from this plant was tested in a study using Wistar rats as model of pyloric ligation and ethanol induced gastric ulcers [91]. The extract showed a gastroprotective function with significant increase in antioxidant levels (CAT and SOD) and anti-secretory activity besides inducing gastric mucosal production. Moreover, the authors suggested that the pharmacological response stated can be attributed to the flavonoid compounds identified in the extract [91].

#### 6.2.37. *Terminalia arjuna*

*Terminalia arjuna* (Roxb.) Wight & Arn. bark contains antioxidant polyphenolics and flavonoids and has been reported to have antibacterial activity [85]. The bark methanol extract (100, 200 and 400 mg/kg) showed marked antiulcer and ulcer healing activities against ethanol, diclofenac sodium and dexamethasone induced ulcer rat models [86]. Moreover, methanol bark extract (100, 200, 300 and 400 mg/kg) showed anti-secretory activity in *H. pylori* lipopolysaccharide-induced gastric ulcer in rats [85]. The authors suggested that the antiulcer effect of *T. arjuna* extract reflects its ability against gastric mucosa damage and its mucosal protective factors [85,86].

#### 6.2.38. *Terminalia belerica*

*Terminalia belerica* Roxb., introduced in Europe and India by Arabs, is a plant used in traditional Ayurvedic medicine, its fruits being one of the three constituents of the important Indian Ayurvedic preparation “Triphala.” The antiulcer activity of 70% methanol extract from *T. belerica* fruits (100, 250, 500, 1000 mg/kg) was evaluated on Wistar rats by employing ethanol, aspirin, cold restraint stress and pylorus ligation ulcer models [87]. This extract was able to suppress ethanol-induced peptic ulcer, at dose of 500 mg/kg, reduced gastric volume, free acidity, total acidity, ulcer index and protein and peptide contents, while increased mucus content in pylorus ligated rats. Moreover, the extract provided protection against aspirin-induced ulcers but not in cold stress restraint model. So, the authors suggested that the possible mechanism of gastric mucosal protection conferred by *T. belerica* methanol extract may be due to reinforcement of the mucosal barrier resistance through protective coating [87].

#### 6.2.39. *Terminalia fagifolia*

*Terminalia fagifolia* Mart. is a medicinal plant used in folk medicine due to its effectiveness in the treatment of gastrointestinal disturbances. Pharmacological activity, including antioxidant effects on gastrointestinal tract of Wistar rats, using the ethanol extract from *T. fagifolia* bark and its aqueous, hydroalcoholic and hexane partition fractions have been evaluated [92]. Paradoxically, the results of this study showed that this plant presents gastroprotective activity and reduces the mucus layer adhered to gastric wall. Ethanol extract had antiulcerogenic activity against ethanol-induced gastric ulcer and ethanol, aqueous and hydroalcoholic partition fractions reduced the mucus layer adhered to gastric wall. Besides to its anti-secretory and antiulcerogenic activities, the plant extract delayed gastric emptying and presented antioxidant activity [92]. 

#### 6.2.40. *Terminalia chebula*

*Terminalia chebula* Retz. is native to southern Asia, from India and Nepal, east to south-western China and south to Sri Lanka, Malaysia and Vietnam. The fruit of this plant is one of the three constituents of the important Indian Ayurvedic preparation “Triphala.” Aspirin, ethanol and cold restraint stress-induced ulcer methods were used in Sprague Dawley rats to assess the antiulcer effects of the hydroalcoholic (70%) extract from *T. chebula* fruits (200 and 500 mg/kg) [89]. The results of this study confirmed the antiulcerogenic potential of the extract, reducing lesion index, total affected area and lesions percentage in aspirin, ethanol and cold restraint stress-induced ulcer models. Moreover, the extracts showed anti-secretory activity in pylorus ligated model, which lead to a reduction in the gastric juice volume, free acidity, total acidity and increased gastric pH [89]. Chebulinic acid was isolated from *T. chebula* fruits and showed anti-secretory and cytoprotective effects on gastric ulcers through the inhibition of H^+^/K^+^-ATPase activity and antioxidant mechanisms [90].

#### 6.2.41. *Argemone mexicana*

*Argemone mexicana* L. is a plant that contains numerous alkaloids and is widely used in traditional medicine. A study carried out to assess the effects of methanol and aqueous extracts from this plant (500–3000 mg/kg), in Wistar rats with duodenal ulceration, concluded that both extracts produced significant activity in cysteamine-induced duodenal ulceration [93].

#### 6.2.42. *Piper betle*

*Piper betle* L. is a plant growing in the tropical humid climate of South East Asia and its leaves are widely consumed as a mouth freshener. The ethanol extract from leaves (200 mg/kg) exhibited protective effects against indomethacin-induced gastric lesions through increasing the antioxidant machinery (SOD and CAT) [95]. Similar results were obtained using ethanol extract at 150 mg/kg after NSAID-induced peptic ulcer in albino rats [94]. Further studies evaluated the role of the major antioxidant constituent present in *P. betle*, allylpyrocatechol, as a gastroprotective agent [96,97,98]. This compound healed indomethacin-induced stomach ulceration in Sprague-Dawley rats by its antioxidant action and ability to form mucus, involving free radical scavenging that protects the gastric mucosa from oxidative damage [96]. Moreover, a study performed in indomethacin-ulcered Swiss albino mice, stated that the anti-inflammatory potential of allylpyrocatechol was mediated by modulation of arginase metabolism, shift of cytokine balance [97] and inhibition of TNF-α, NF-κB and JNK pathways [98].

#### 6.2.43. *Ficus religiosa*

*Ficus religiosa* L. is a plant species belonging to the Moraceae family that has been recently studied, namely its phytochemicals, as a potential H2 receptor antagonist using molecular docking approach and lanosterol and α-amyrin acetate were found to have higher stability during simulation studies. Hence, these compounds may be suitable therapeutic agents on PU treatment, acting as H2 receptor antagonist [99]. 

#### 6.2.44. *Scutia buxifolia*

Boligon et al. [29] reported that the stem back extract of *Scutia buxifolia* Reissek exhibited antiulcerogenic and protective effects on gastric mucosa against ethanol-induced oxidative injury in experimental model of gastric ulcer, this protection being attributed to the antioxidant properties of the constituent phenolic compounds of the plant.

#### 6.2.45. *Ziziphus jujuba*

*Ziziphus jujuba* Miller is a plant species belonging to Rhamnaceae family, commonly used in Persian folk medicine for the treatment of gastrointestinal diseases, such as ulcers [2], and both fruits and stem are employed to treat digestive disorders. Fruits possess antitussive, laxative and hypotensive properties, while the stem back and leaves could cure wounds and PU. Its bark, has been traditionally employed by Iranian healers to treat digestive disorders and gastric ulcers. The effect of the aqueous extract from *Z. jujuba* stem bark (100, 200 and 400 mg/kg) against acidified ethanol-induced gastric ulcers in albino Wistar rats, as well as its anti-*H. pylori* activity was tested by disc diffusion assay [2]. As the main findings of this study, the extract exhibited antiulcer potential through protecting gastric mucosa and anti-*H. pylori* activity. Moreover, the authors proposed that the flavonoids present in the stem bark extract may be responsible from the observed effects due to increased gastric wall mucus; in turn, the mechanism of gastric mucosal protection may be due to the enforcement of mucosal barrier through a protective coating, in addition to the antioxidant activity [2]. Beyond that, anti-inflammatory, antimicrobial, antisteroidogenic and antioxidant properties have also been attributed to this plant. 

#### 6.2.46. *Cecropia glaziovii*

*Cecropia glaziovii* Snethl. is a fast-growing and short-lived tree native to tropical Central and South America regions, which is used in folk medicine. A study reported the antiulcer and anti-secretory activities of the aqueous extract from this plant (0.5, 1.0 and 2.0 mg/kg) on gastric acid secretion of pylorus-ligated Swiss albino mice [100]. Furthermore, the authors isolated the butanolic fraction of the aqueous extract and suggested that the main compounds isolated (e.g., catechins, procyanidins and flavonoids) are responsible by the decrease in rabbit gastric H^+^/K^+^-ATPase activity in vitro, proportionately to the concentration used (IC_50_ = 58.8 µg/mL). With these findings, the authors concluded that *C. glaziovii* extract constituents inhibited the gastric proton pump provoking the anti-secretory and antiulcer activities [100].

#### 6.2.47. *Osyris quadripartita*

*Osyris quadripartita* Salzm. ex Decne is a plant native of Africa, southwestern Europe and Asia, commonly known as wild tea and widely used in Ethiopian folk medicine. A study evaluated the antiulcer activity of 80% methanol leaf extract from *O. quadripartita* in Wistar albino rats, using pylorus ligation-induced and ethanol-induced models, both applying a single (100, 200, 400 mg/kg) and repeated dosing (200 mg/kg for 10 and 20 days) approaches [101]. The extract reduced gastric ulcer index in both models at 400 mg/kg dose, which is comparable to the standard drugs, ranitidine (50 mg/kg) and sucralfate (100 mg/kg). Moreover, the study showed that the extract exerted both dose- and time-dependent antiulcer effects in both models. Additionally, the oral median lethal dose (LD_50_) was estimated to be higher than 2000 mg/kg and where secondary metabolites, including flavonoids, tannins and saponins were detected in the extract [101].

#### 6.2.48. *Anacardium occidentale*

*Anacardium occidentale* L.—commonly known as cashew tree—is native to Brazil but presently cultivated in many regions of the world. It is used by folk medicine in South America, namely its leaves, in the form of tea. The hydroethanolic extract from *A. occidentale* leaves inhibited gastric lesions induced by HCl/ethanol in Wistar rats [104]. Further, a dose-response effect study showed that the ED_50_ was 150 mg/kg. The chemical analysis of the extract detected, respectively, 35.5% and 2.6% of total phenolics and flavonoids. The use of aqueous leaf extract seemed not to involve the lowering of acid secretions [102]. Also, the use of percolated extract from *A. occidentale* at 200 mg/kg did not provoke any ulcerogenic consequence in rat's stomach and at doses of 300, 400 and 800 mg/kg presented less gastric ulcerogenity than similar doses of indomethacin [103]. 

In a study designed to investigate the effect of anacardic acids in gastroprotection against ethanol-induced gastric damage [105], the authors verified the existence of a gastroprotective role conferred by anacardic acids and suggested that this effect could possibly be mediated by an antioxidant mechanism, as the anacardic acids were able to restore the non-protein sulfhydryls, CAT, SOD and nitric oxide levels [105]. Moreover, it was also proposed that anacardic acid may activate capsaicin-sensitive gastric afferents, stimulate endogenous prostaglandins and nitric oxide and open K^+^ ATP channels [105].

#### 6.2.49. *Anacardium humile*

*Anacardium humile* St. Hill, namely its leaves and bark infusions, are used in folk medicine. The crude methanol extract obtained from *A. humile* leaves (250, 500 and 1000 mg/kg) has shown to possess antiulcerogenic activity against ethanol and piroxicam-induced gastric lesions in Wistar rats and Swiss albino mice, respectively [106]. The gastroprotective activity of the ethyl acetate extract from *A. humile* leaves (50, 100, 200 mg/kg) was also evaluated in another study using ethanol-induced acute gastric mucosal injury as a model, in Wistar rats [107]. The results showed that the extract exhibited gastroprotection against ethanol, involving mechanisms based on its capacity to strengthen defensive factors and raising mucus and PGE2 levels, with participation of nitric oxide and sulfhydryl groups to prevent or to attenuate the ulcer process [107].

#### 6.2.50. *Spondias mombin*

*Spondias mombin* L. is a fructiferous tree found in the rain forest and coastal areas of Nigeria, where it has found usage in folk medicine for the treatment of many diseases due to its bioactive substances including tannins, saponins, flavonoids, phenolics and anthraquinone glycosides [108]. In fact, teas and infusions from its leaves and flowers have been broadly used as anti-inflammatory and analgesic against stomachache and discomfort. Even, a recent study has shown that *S. mombin* leaf extracts was capable of ameliorating indomethacin-induced gastric ulceration via antioxidant and proton pump inhibition mechanisms [109].

#### 6.2.51. *Toona ciliata*

*Toona ciliata* M. Roem is a tall tree with colored wood, widely distributed in the Himalayan region. Ethanol heart wood extract of *T. ciliata* (300 mg/kg) was evaluated for its antiulcer activity against aspirin plus pylorus ligation induced gastric ulcer, HCl-ethanol induced ulcer and water immersion stress induced ulcer in Wistar albino rats [110]. The authors found that the studied extract decreased the incidence of ulcers in the three models tested and concluded that *T. ciliata* heartwood extract exerted antiulcerogenic effects through anti-secretory, cytoprotective and proton pump inhibitory mechanisms [110].

#### 6.2.52. *Bryophyllum pinnatum*

*Bryophyllum pinnatum* (Lam.) Oken is a medicinal plant used in India, Africa, China and tropical America that contains different groups of phytoconstituents, among them flavonoids, phenolic acids, alkaloids and terpenoids. In Wistar rats, the isolated mucilage (500 mg/kg) and the aqueous extract (500 and 750 mg/kg) from whole plant exerted both potent gastroprotective effects against ethanol-induced ulcer model [111]. The results of this study showed a dose-dependent protection as ascertained by the reduction of ulcer area in gastric wall, as well as reduction or inhibition of edema and leucocyte infiltration of sub-mucosal layers [111]. In another study performed using methanol leaf extract from *B. pinnatum*, a pronounced gastroprotection was observed on aspirin-induced ulcer in pylorus-ligated rats and histamine-induced duodenal lesions in guinea pigs [112]. 

#### 6.2.53. *Aframomum pruinosum*

*Aframomum pruinosum* Gagnep. and even other species from the *Aframomun* genus, are traditionally used in Cameroon to cure gastritis. A methanolic extract from *A. pruinosum* seeds (125, 250 and 500 mg/kg) was evaluated as antiulcerogenic agent using two different models, NSAID- and *H. pylori*-induced gastric lesion in rats [113]. This study concluded that the extract from this plant possessed a moderate anti-*Helicobacter* and antiulcer activities, with a cytoprotective action due to the increase in mucus production stimulated by the endogenous prostaglandins and nitric oxide generation mechanisms [113].

### 6.3. Phytochemicals with Antiulcerogenic Activity

**Figure 1:**
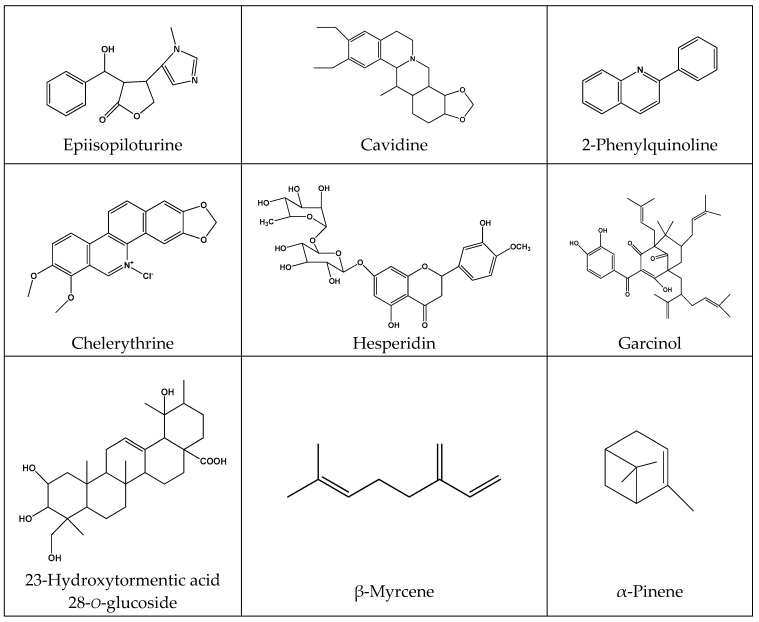

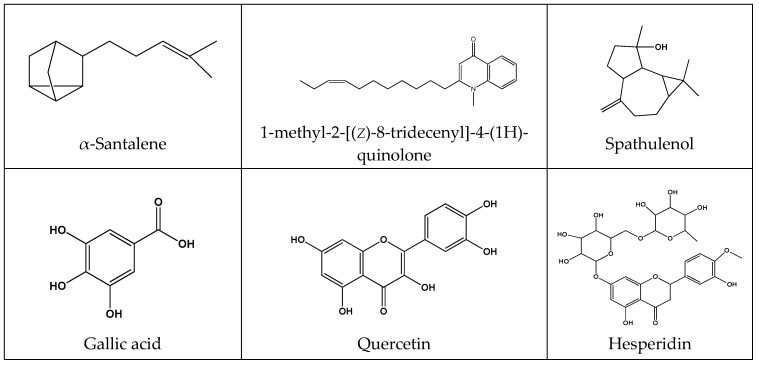
Structure of some phytochemicals evaluated as antiulcer agents.

Medicinal plants are safe, cheap, effective and available source of biologically active molecules [122,123,124,125,126,127,128,129,130,131,132,133]. Plants biological activity is mainly related to the presence of plant secondary metabolites (PSM), which have a specific function and role [134,135,136,137]. Indeed, a wide pool of phytochemicals including tannins, flavonoids, alkaloids, terpenoids and phenolic glycosides have been reported to be responsible for the observed gastroprotective and antiulcerogenic properties of the various plants used in PU management and this suggests plants and their bioactive phytochemicals as upcoming viable sources of antiulcer agents. Furthermore, the therapeutic benefits of plant extracts may be attributed both to a single component or even to the combined action of a mixture of phytoconstituents [138,139,140,141,142,143,144,145,146,147]. Figure 1 shows the most common and widely used PSM with antiulcer activity, which majorly includes alkaloids, flavonoids, phenolic acids and essential oils.

There are four main classes of drugs used in antiulcer therapy: antacids; H2 receptor antagonists; proton pump inhibitors; and potassium-competitive acid blockers [148]. Numerous PSM have been reported to display antiulcer effects through different mechanisms of action in many experimental models of ulcers that are induced by ethanol, acetic acid, NSAIDs, stress, *H. pylori* and so on. Indeed, PSM exert antiulcer activity through multiple mechanisms; predominantly via antioxidant, anti-inflammatory, antimicrobial, anti-secretory, anticholinergic and cytoprotective effects [149]. The phytochemical-related modes of action, dose and experimental models of ulcer are summarized in Table 3.

### 6.4. Principle Components and Their Antiulcer Inhibitory Effects

#### 6.4.1. Alkaloids

Alkaloids represent a group of natural products that are nitrogen containing PSM, which display a considerable antiulcer activity. Falcao and et al. [178] reviewed gastric and duodenal antiulcer activity of sixty-one alkaloids; fifty-five of them exerted antiulcer effects. One important advantage of alkaloids compared to others PSM is that they have good solubility in acidic medium (stomach juice).

Epiisopiloturine hydrochloride, an imidazole alkaloid isolated from *Pilocarpus microphyllus* leaves, protects against naproxen-induced gastrointestinal damage in rats by reducing pro-inflammatory cytokines and oxidative stress and increasing gastric mucosal blood flow. Pretreatment with epiisopiloturine prevented naproxen-induced macro and microscopic gastric damages with maximal effects at 10 mg/kg [154]. Cavidine, a major alkaloid compound isolated from Co*rydalis impatiens* reduced gastric injuries in mice with ethanol-induced acute gastric ulcer at 10 mg/kg. Also, cavidine treatment resulted in increased mucosa GSH, SOD and PGE2 levels, while decreased IL-6 and TNF-α levels [152]. Uleine isolated from *Himatanthus lancifolius* resulted in increasing GSH levels and an antioxidant response and a decrease in H^+^/K^+^-ATPase activity at the pylorus ligature-induced ulcer in rats at 30–82 mg/kg. Alkaloids from *Mahonia bealei* possess anti-H^+^/K^+^-ATPase effects on pyloric ligation-induced gastric ulcer in rats at 18.6 mg/kg/day. Besides, 2-phenylquinoline effects were attributed to SOD and glutathione-*S*-transferase (GST) normalization activity and reduction in lipid peroxide (LPO) and TNF-α levels in the gastric mucosa from rats with gastric ulcer induced by 60% ethanol/0.03 M hydrochloric acid (HCl) and indomethacin [151]. Chelerythrine reduced myeloperoxidase activity and nitric oxide concentration, pro-inflammatory IL-6 and TNF-α levels in ethanol-induced gastric ulcer mice at 1.5–10 mg/kg [153]. Also, quinolone alkaloids from *Evodia rutaecarpa* have shown highly selective antibacterial activity against *H. pylori,* the minimum inhibitory concentration (MIC) found being 0.05 µg/mL [150]. Still, an alkaloid-rich fraction extract from *Tylophora conpicua* was able to decrease histamine insulted gastric acid secretion in rats [155].

#### 6.4.2. Terpenes and Terpenoids

Monoterpene β-myrcene isolated from *Citrus aurantium* decreased gastric and duodenal lesions, increased gastric mucus production and mucosal MDA levels, GPx and GR levels and decreased SOD activity in experimental ulcers models induced by ethanol, NSAIDs, stress, *H. pyroli*, ischemia reperfusion injury and cysteamine at 7.5 mg/kg [162]. α-Pinene also confirmed antibacterial effects on metronidazole-resistant *H. pylori* at ethanol-induced gastric ulcer, with an EC_50_ value of 12.32 mg/kg [158]. α-Pinene-rich essential oil (50.8%) increased gastric mucus production and induced PGE2 levels [159]. The volatile oil of *Cedrus deodara* significantly reduced ulcers at a dose of 100 mg/kg, which justifies the traditional usage of this herb to treat peptic ulcers. Ulcer inhibition of 100 mg/kg *Cedrus deodara* and 20 mg/kg of rabeprazole was, respectively, 41.5% and 67.7% [179]. α-Santalene rich essential oil of *Gallesia integrifolia* evidenced potent gastroprotective and curative effects in vivo and in vitro experimental models, which is probably due to its antioxidant, nitrergic, mucogenic, anti-secretory and anti-inflammatory effects [161]. On the other hand, the essential oil of *Croton rhamnifolioides* with major components spathulenol (22.5%) and 1,8-cineole (18.3%) exhibited antiulcer activity by modulation of opioid receptors and nitric oxide [163]. Triterpenoids 23-hydroxytormentic acid 28-*o*-glucoside isolated from *Rubus coreanus* increased SOD and GPx activities in rats with ulcer induced by combination of ethanol and sodium salicylate [157].

#### 6.4.3. Flavonoids

Flavonoids are natural antioxidants present in different kinds of fruits and vegetables, possessing a characteristic C6-C3-C6 carbon skeleton structure. Recent studies indicated that flavonoid shows a wide range of pharmacological activities, including as antiallergic, anti-inflammatory, antimicrobial, anti-cancer, antidiarrheal and antiulcer. Due to the presence of a hydroxyl group(s) in their aromatic ring(s), they possess antioxidant activity. Quercetin, rutin and kaempferol are widespread in the plant kingdom. They inhibited the mucosal content of platelet-activating factor in rats with gastric damage produced by acidified ethanol [172]. Rutin and quercetin isolated from *Piper umbellatum* L. showed antiulcer effect by exerting antioxidant, anti-secretory, anti-inflammatory and mucosa regenerative activities [173]. Hesperidin increased GSH and mucin levels and prevented oxidative cell injury in indomethacin and hypothermic restrain stress-induced ulceration models in rats [171]. *Caryocar coriaceum* extract, with gallic acid, chlorogenic acid, caffeic acid, rutin and quercetin as major constituents, exhibited antiulcer activity through opioid and α_2_-adrenergic receptors and primary afferent neurons sensitive to capsaicin in gastric ulcers induced by ethanol, acidified ethanol, acetic acid or indomethacin [169]. Anthocyanins extracted from *Rubus coreanus* have shown antiulcer effect in association with the regulation of the matrix metalloproteinase-2 activity, preventing lipid peroxidation and even increasing CAT, SOD and GPx activities [164]. Garcinol suppressed superoxide anion, hydroxyl radical and methyl radical in rats with acute ulceration stress induced by indomethacin and water immersion [170].

#### 6.4.4. Saponins

Probably due to the presence of antioxidant saponins, the aqueous extract from *Bauhinia purpurea* leaf exhibited in vivo antiulcer activity, which confirm the traditional uses of *B. purpurea* in the treatment of ulcers [180].

#### 6.4.5. Phenolic Acids

*p*-Coumaric acid elicited antioxidant activity by attenuating ulcers due to elevated MDA levels, reduced GSH levels and decreased SOD, CAT, GPx and GR activities [174]. Gallic acid (1 mg/mL) showed high in vitro inhibitory effects against two *H. pylori* strains [167]. Ellagic and gallic acids presented a prominent antiulcer action related to prostaglandins and nitric oxide/cyclic guanosine monophosphate pathway [165]. A synergistic antiulcer activity using gallic acid and famotidine combination was observed against aspirin plus pyloric ligation induced ulcer in rats. Combination treatment resulted in increased levels of SOD, CAT, GR and glucose-6-phosphate dehydrogenase, while decreased lipid peroxidation and myeloperoxidase in gastric tissues [168].

#### 6.4.6. Tannins

Tannins fraction of *Mouriri pusa* augmented cell proliferation, anti-inflammatory activity by reducing COX-2 levels, enhanced angiogenesis and increased mucus secretion [176]. Ellagitannin-rich fraction increased GSH and SOD levels in rats with ethanol-induced gastric ulceration model [175]. Hydroalcoholic extract from *Persea major* bark exerted antiulcer effects in rodents through empowering gastric protective factors. The main compounds found in the hydroalcoholic extract of *P. major* (HEPM) were polyphenols, such as condensed tannins, flavonoids heterosides derivatives from quercetin and kaempferol. HEPM (300 mg/kg) prevented gastric lesions induced by ethanol or indomethacin in rats by 58.98% and 97.48%, respectively, compared to the vehicle group (148 mm^2^ and 12 mm^2^, respectively) [181].

#### 6.4.7. Fatty Acids

Oleic acid accelerated ROS and nitric oxide (NO) synthesis and reduced oxidative damage; it also reduced inflammatory cells infiltration and TNF-α expression in mice using ischemia-reperfusion-induced skin injury model [177].

## 7. Safety of Plant Products Used as Antiulcerogenic Agents

In an attempt to find an effective treatment for various diseases, the modern medicine turns to traditional medicine. There are decades of using specific plants or certain plants parts in the treatment of several health conditions throughout the world. Many side effects because of using conventional antiulcer drugs has shifted the search for new drugs to folk and traditional medicines. Indeed, there is an effort in pharmacology to confirm the real benefits of traditionally used plants in antiulcer therapy and to identify active compound(s) responsible for their positive effects. The new modern approach with improved technology is unthinkable without the use of laboratory animals. Nonetheless, there are few animal models developed in ulcers research, usually including rats. All ethical principles for using of animals in experiments should be followed and protocols approved by the Institutional Ethics Committee.

The assessment of the acute gastroprotective activity of plant extracts is generally performed using two different animal models of gastric ulcer: ethanol-induced and indomethacin (a non-steroidal anti-inflammatory agent)-induced gastric ulcer. On the other hand, the evaluation of the chronic gastroprotective activity is usually performed on animals with gastric ulcer induced by acetic acid.

The growing interest in herbal medicines is partly derived from the results of many animal studies, indicating that plant extracts have lower toxicity than synthetic drugs [182]. However, for using specific plants extracts as antiulcer agents, the data of acute and chronic toxicity should be obtained. Acute toxicity studies, also known as single dose studies and chronic (or sub-acute) studies, also named repeated dose studies, are of crucial importance in case of testing extracts from plants which include a high number of different substances. There are few recommendations for toxicity studies and how to be performed, being one of them described by Organization for Economic Cooperation and Development (OECD) [183]. Rats and mice are usually used to the in vivo assessment of drugs safety. During the examination of plants biological activities, the first step that should be provided is the detection of acute toxicity. In acute toxicity assay, single dose limit is 2000 mg/kg or 5000 mg/kg body weight and if the substance is not lethal on acute administration of 5000 mg/kg body weight, according to toxicologists through the world, that substance is essentially considered non-toxic [184]. Indeed, the benefits of using plant products as antiulcer agents due to their low toxicity are clearly evident, particularly in cases where a single dose of 5000 mg/kg body weight do not evoke any change, as was observed in mice treated with an hydroalcoholic extract obtained from *Serjania marginata* Casar. (fam. Sapindaceae) [185] or with an ethanol extract of *Crassocephalum vitellinum* (fam. Asteraceae) [186], both plants with proven antiulcer activity. The results of acute toxicity are usually expressed as the required dose in g/kg body weight which cause death in 50 % of the animals tested (LD_50_) [187]. Notwithstanding, before proceeding to the development of a pharmaceutical formulation, long-term in vivo chronic toxicity in animal models should be assessed.

The experiments performed in animal models of gastric ulcer confirm the gastroprotective and healing properties of many herbs traditionally used in folk medicine. One of them is the ethanol extract from *Vernonia condensata* (Asteraceae) [39], where the treatment in rats with 300 mg/kg, p.o., twice daily for 7 days, did not produce any sign of acute toxicity, none of the animals died and no significant changes in organ weight were stated. Also, the oral administration of hydroalcoholic extract from *Maytenus robusta* (Celasteraceae) over 7 days twice a day at dose of 10 mg/kg, in rats, did not produce any sign of toxicity [49]. However, in a previous study, the same plant extract indicated in vivo genotoxicity in mammalian cells at higher doses (250 or 500 mg/kg), while there was no genotoxic effect at 50 mg/kg [188]. Aqueous and chloroform extracts of *Bauhinia purpurea* (Fabaceae) [184,189] and ethanol extract of *Parkia speciosa* (Fabaceae) [62] at the single oral dose of 5000 mg/kg did not produce any sign of toxicity, behavioral abnormality or mortality in rats for the next 14 days, while its antiulcer activity was confirmed in all used in vivo models of gastric ulcer. Similar results were obtained in acute toxicity test of *Clausena excavata* (Rutaceae) methanol extract, in rats, using a single dose (2000 mg/kg or 5000 mg/kg, p.o.); the oral LD_50_ higher than 5 g/kg body weight indicated that it is relatively safe and, therefore, considered to be non-toxic [190]. However, in this study, it was observed that body weight was lower in the group treated with high doses of extract.

The acute toxicity test carried out in rats at two doses (1000 mg/kg or 2000 mg/kg) of ethyl acetate extract from *Annona muricata* (Annonaceae) leaves showed the safety of this plant and that the oral LD_50_ is higher than 2 g/kg, which correlated with the previous results obtained in mice (where LD_50_ was 1.67 g/kg) [191]. No signs of toxicity were observed in rats after using a single dose (2000 mg/kg) of hydroalcoholic extracts from *Brassica oleracea* (Brassicaceae) [192] and *Caesalpinia sappan* (Caesalpiniaceae) [57], confirming even its in vivo gastroprotective effects in different gastric ulcer models. Although most studies on the acute oral toxicity of plant extracts show no signs of toxicity, in the case of the dichloromethane fraction of *Piper tuberculatum* (Piperaceae) fruit [193], following administration of an oral dose of 5000 mg/kg, in mice, respiratory changes, pilomotor erection and reduction in locomotion and passivity were stated 10 min after treatment, resulting in animal death after 4 h. The same dose of this extract administrated i.p. triggered similar symptoms, which appeared earlier and resulted in animal death after 30 min. LD_50_ were 1.62 g/kg p.o. and 0.26 g/kg i.p.

While there is a certain number of data that involves acute toxicity studies of plant products used as antiulcer agents, the information on chronic or sub-acute toxicity studies are missing or sparsely performed. One of the exceptions in this field are data obtained from aqueous extract of *Achillea millefolium* (Asteraceae), popularly known as yarrow. It has been shown that the aqueous extract of yarrow has protective effects in ethanol and indomethacin induced gastric lesions and healing properties in acetic acid-induced chronic gastric lesions. In the study of chronic toxicity, tests were performed in both genders of Wistar rats for 28 and 90 consecutive days [194]. Besides, a satellite group of animals sacrificed 30 days after the end of treatments was also included. All rats survived period after the 90-day repealed oral exposure to aqueous extract of yarrow. Slight changes in liver weight, cholesterol, HDL-cholesterol and glucose levels were observed, though these changes were not correlated with dose applied or treatment duration. There was no change in body mass, clinical and behavioral patterns in treated and satellite groups, which indicated that the extract is non-toxic. Based on these findings, it was stated that a very long chronic exposure does not have toxicological risk. Also, performing a Hippocratic test by acute administration of yarrow aqueous extract in dose of up to 10000 mg/kg (p.o.) and 3000 mg/kg (i.p.) did not cause animals death.

Subacute toxicity evaluation was carried out using the hydroethanolic extract of *Sedum dendroideum* (Crassulaceae), which showed gastroprotective effects [195]. Wistar rats of both genders received the most effective hydroethanolic extract dose (50 mg/kg) after acetic acid-induced gastric ulcer, over 14 days. Animals were daily monitored but no significant differences were observed in body mass index, organs weight, biochemical parameters, behavioral disorders, feeding pattern and water intake [195].

One of the possible approaches in gastric ulcer treatment is the combination of synthetic drugs with plant products, which alleviates the side effects of conventional drugs for long-term treatment. The methanol extract of *Bambusa arundinacea* (Gramineae) exhibited antiulcer activity and toxicity studies carried out in Swiss Albino mice of both genders showed that there is no significant change in autonomic responses, though these animals were less active compared to the control group [196]. Calculated LD_50_ was 2.55 g/kg (p.o.) and 1.81 g/kg (i.p.). Otherwise, curiously, the combination of *B. arundinacea* methanol extract and phenylbutazone (a NSAID) produced a more powerful anti-inflammatory agent, with fewer toxic effects and without ulcer formation.

Based on this data, the examination of the main constituents of plant extracts could also provide an approach in investigating possible antiulcerogenic effects of plant products and performing toxicity studies with specific substances. Interestingly, in a study performed using the methanol fraction of *Euphorbia umbellata* (Euphorbiaceae) bark, it was concluded that the observed antiulcerogenic properties depended on polyphenols, primarily ellagic and gallic acids derivatives and flavonols, though there are no toxicity studies [165]. Also, oleanolic acid, a triterpene widely distributed in plants, was shown to improve chronic gastric lesion healing in rats, with low toxic effects [197].

## 8. Conclusions and Future Perspectives

The overall findings shared in this report clearly stress that plant products represent a rich source of bioactive molecules with antiulcer potential. Moving from traditional uses to preclinical studies, the efficacy of certain herbal remedies has been substantially investigated by in vitro and even in vivo studies, and, in some cases, their activity has been ascribed to specific classes of phytochemicals, such as alkaloids, tannins, simple phenols and polyphenols (particularly flavonoids).

Analysis of literature data indicated that phytochemicals are natural, safe and effective resources that can be used in the prevention and even treatment of ulcers. However, the paucity of human studies, at the top of the evidence-based medicine pyramid, slows down these promising findings, thus requiring, in the near future, more clinical trials to support and even to validate the myriad of preclinical data.

## Figures and Tables

**Table 1:** Main drug classes used in peptic ulcer management.

Drug Classes	Characteristics	Types	Reference
Antacids	Help in neutralizing gastric acid, reducing acid delivery in duodenum and pepsin activity, besides to bind bile acids	Calcium and magnesium carbonates, aluminum hydroxide and magnesium trisilicate	[21,22,23]
Anti-secretory agents	Reduce gastric acid secretion, help relieve ulcer pain and stimulate ulcer healing, inhibit *H. pylori* growth and proliferation in gastric tissues	Histamine H2-receptor antagonist (cimetidine, famotidine, nizatidine and ranitidine), proton pump inhibitors (esomeprazole, lansoprazole, omeprazole, pantoprazole and rabeprazole)	[24]
Cytoprotective agents	Reduce/prevent gastric mucosal damage (increase mucus and bicarbonate secretion, strengthen gastric mucosal barrier, decrease gastric motility, increase blood flow to gastric mucosa, increase prostaglandins and sulfhydryl biosynthesis, scavenge free radicals, stimulate cell growth and repair and decrease leukotrienes release)	Prostaglandins, fatty acids, sulfhydryl compounds, aluminum-containing antacids, sucralfate, bismuth chelate and liquorice	[25,26]

**Table 2:** Plant extracts with antiulcerogenic activity.

Order	Family	Binomial Name	Mechanism of Gastroprotection	Reference
Apiales	Apiaceae	*Centella asiatica*	Wound healing, mucus production, antioxidant, anti-inflammatory	[30,31]
Asterales	Asteraceae	*Baccharis dracunculifolia*	Wound healing, antioxidant, mucus production	[32,33]
		*Baccharis trimera*	Wound healing, anti-secretory, antioxidant	[34,35,36]
		*Hieracium gymnocephalum*	Wound healing, anti-inflammatory	[37]
		*Tanacetum larvatum*	Wound healing, anti-inflammatory, antioxidant	[38]
		*Vernonia condensata*	Wound healing, inhibition of gastric secretion, antioxidant, mucus production, cytoprotective	[39,40]
		*Solidago chilensis*	Wound healing, antioxidant, anti-secretory, mucus production	[41,42]
Boraginales	Boraginaceae	*Cordia dichotoma*	Wound healing, antioxidant, anti-inflammatory	[43]
Brassicales	Capparaceae	*Capparis zeylanica*	Wound healing	[44]
	Moringaceae	*Moringa oleifera*	Wound healing, serotonin release, anti-secretory, cytoprotective, anti-inflammatory	[45,46,47]
	Salvadoraceae	*Salvadora indica*	Wound healing, cytoprotective	[48]
Celastrales	Celastraceae	*Maytenus robusta*	Wound healing, mucus production, antioxidant, anti-inflammatory, cytoprotective, gastroprotective, anti-secretory	[27,28,49,50]
Cucurbitales	Cucurbitaceae	*Momordica cymbalaria*	Wound healing, anti-secretory	[51]
		*Mukia maderaspatana*	Wound healing, antioxidant, anti-inflammatory, mucus production	[52]
Cyatheales	Cibotiaceae	*Cibotium barometz*	Wound healing, antioxidant, HSP70 up-regulation, Bax protein down-regulation, mucus production	[53,54]
Cyperales	Cyperaceae	*Cyperus rotundus*	Wound healing, antioxidant activity, anti-inflammatory	[55,56]
Fabales	Caesalpinieae	*Caesalpinia sappan*	Wound healing, antioxidant, cytoprotective, anti-inflammatory	[57]
	Fabaceae	*Archidendron jiringa*	Wound healing, mucus production, antioxidant	[58]
		*Alhagi maurorum*	Antioxidant, antiapoptotic	[59]
		*Cassia sieberiana*	Wound healing, cytoprotective, antioxidant, anti-inflammatory	[60,61]
		*Parkia speciosa*	Wound healing, mucus production, antioxidant, anti-inflammatory, HSP70 up-regulation, Bax protein down-regulation	[62]
		*Tamarindus indica*	Wound healing, anti-secretory	[63]
Gentianales	Rubiaceae	*Morinda citrifolia*	Wound healing, anti-secretory, antioxidant, anti-inflammatory	[64]
Lamiales	Bignoniaceae	*Kigelia africana*	Wound healing, antioxidant	[65]
	Boraginaceae	*Cordia verbenacea*	Wound healing, antioxidant, cytoprotective	[66]
	Acanthaceae	*Barleria lupulina*	Anti-inflammatory, wound healing, anti-secretory	[67,68]
		*Eremomastax speciosa*	Wound healing, anti-secretory, mucus production, antioxidant, cytoprotective	[69,70,71]
	Lamiaceae	*Calamintha officinalis*	Wound healing, antioxidant	[72]
		*Hyptis suaveolens*	Wound healing, cytoprotective, anti-inflammatory	[73,74]
		*Tectona grandis*	Wound healing, inhibition of gastric secretion	[75,76]
Magnoliales	Myristicaceae	*Myristica malabarica*	Wound healing, anti-inflammatory, angiogenesis, cytoprotective	[77,78,79,80]
Magnoliopsida	Talinaceae	*Talinum portulacifolium*	Wound healing, anti-secretory	[81]
Malpighiales	Calophyllaceae	*Mammea americana*	Wound healing, anti-secretory	[82]
	Hypericaceae	*Cratoxylum arborescens*	Wound healing, anti-*H. pylori*, anti-secretory, mucus production, antioxidant, antiapoptotic, anti-inflammatory, cytoprotective	[83,84]
Myrtales	Combretaceae	*Terminalia arjuna*	Wound healing, anti-*H. pylori*, anti-secretory	[85,86]
		*Terminalia belerica*	Wound healing, anti-secretory, mucus production	[87]
		*Terminalia catappa*	Wound healing, anti-*H. pylori*, mucus production, anti-inflammatory, cytoprotective	[88]
		*Terminalia chebula*	Wound healing, anti-secretory, cytoprotective	[89,90]
		*Terminalia coriacea*	Wound healing, anti-secretory, mucus production, antioxidant	[91]
		*Terminalia fagifolia*	Wound healing, anti-secretory, antioxidant	[92]
Papaverales	Papaveraceae	*Argemone mexicana*	Wound healing	[93]
Piperales	Piperaceae	*Piper betle*	Wound healing, antioxidant, mucus production, anti-inflammatory	[94,95,96,97,98]
Rosales	Moraceae	*Ficus religiosa*	Wound healing, inhibition of gastric secretion	[99]
Santalales	Rhamnaceae	*Scutia buxifolia*	Wound healing, antioxidant	[29]
		*Ziziphus jujuba*	Anti-*H. pylori*, mucus production, antioxidant	[2]
	Urticaceae	*Cecropia glaziovii*	Wound healing, anti-secretory	[100]
	Santalaceae	*Osyris quadripartita*	Wound healing	[101]
Sapindales	Anacardiaceae	*Anacardium occidentale*	Wound healing, antioxidant, anti-inflammatory, cytoprotective	[102,103,104,105]
		*Anacardium humile*	Wound healing, mucus production, anti-inflammatory, cytoprotective	[106,107]
	Meliaceae	*Spondias mombin*	Wound healing, antioxidant, anti-inflammatory, inhibition of gastric secretion	[108,109]
		*Toona ciliata*	Wound healing, anti-secretory, cytoprotective	[110]
Saxifragales	Crassulaceae	*Bryophyllum pinnatum*	Wound healing, anti-inflammatory	[111,112]
Zingiberales	Zingiberaceae	*Aframomum pruinosum*	Wound healing, anti-*H. pylori*, mucus production, anti-inflammatory, cytoprotective	[113]

**Table 3:** Antiulcer activity of phytochemicals.

Phytochemicals	Plant Source	Model	Dose/Results	Mode of Action	Reference
**Alkaloids**					
1-methyl-2-[(*z*)-8-tridecenyl]-4-(1*H*)-quinolone and 1-methyl-2-[(*z*)-7-tridecenyl]-4-(1*H*)-quinolone	*Evodia rutaecarpa*	In vitro antibacterial activity against *H. pylori*	0.05 μg/mL	Highly selective activity against *H. pylori*	[150]
2-Phenylquinoline	*Galipea longiflora*	Gastric ulcer induced by 60% ethanol/0.03 M HCl, indomethacin-induced acute lesions in rats	10–100 mg/kg	SOD and GST activity normalization, increased GSH and reduced LPO and TNF-α levels in gastric mucosa	[151]
Cavidine	Co*rydalis impatiens*	Ethanol-induced acute gastric ulcer in mice	10 mg/kg	Increased mucosa GSH, SOD and PGE2 levels, decreased IL-6 and TNF-α levels	[152]
Chelerythrine	Papaveraceae and Rutaceae family	Ethanol-induced gastric ulcer in mice	1.5, 10 mg/kg	Reduced myeloperoxidase activity, IL-6 and TNF-α levels and inhibited NO	[153]
Epiisopiloturine	*Pilocarpus microphyllus*	Naproxen-induced gastrointestinal damage in rats	10 mg/kg	Reduced pro-inflammatory cytokines, oxidative stress and increased gastric mucosal blood flow	[154]
Uleine	*Himatanthus lancifolius*	Ethanol-induced acute gastric ulcer and pylorus ligature-induced ulcer in rats	30, 82 mg/kg	Increased GSH level, antioxidant response and decreased H^+^/K^+^-ATPase activity	[155]
Alkaloid fraction extract	*Tylophora conpicua*	Gastric acid secretion and ulceration in rat	40 mg/kg	Decreased histamine insulted gastric acid secretion	[155]
Alkaloid fraction (columbamine, jatrorrhizine, palmatine and berberine)	*Mahonia bealei*	Pyloric ligation-induced gastric ulcer in rats	18.6 mg/kg	Anti-H^+^/K^+^-ATPase anti-gastrin effects	[156]
**Terpenes and Terpenoids**					
23-hydroxytormentic acid 28-*o*-glucoside and its aglycone	*Rubus coreanus*	Gastric ulcer induced by oral administration of ethanol + sodium salicylate	10, 30 mg/kg	Increased SOD and GPx activity	[157]
α-Pinene	*Pistacia atlantica*	Ethanol-induced gastric ulcer	12.32 mg/kg (EC_50_)	Antibacterial activity on metronidazole-resistant *H. pylori*	[158]
α-Pinene (50.8%), cineole (20.3%), β-pinene (18.3%)	*Hyptis spicigera*	Ethanol and NSAIDs rodent models	100 mg/kg	Increased gastric mucus production and induced PGE2	[159]
α-Pinene (13.4%), 1,8-cineole (18%), camphor (32.8%), β-caryophyllene (12.9%)	*Hyptis crenata*	Gastric ulcer induced by oral administration of absolute ethanol or indomethacin	30, 100, 300 mg/kg	Accelerated gastric emptying effect and reduced oxidative damages	[160]
α-Santalene	*Gallesia integrifolia*	In vivo and in vitro experimental models	80 mg/kg	Gastroprotective and curative effects, probably due to antioxidant, anti-inflammatory, anti-secretory, mucogenic and nitrergic and activity	[161]
β-Myrcene	*Citrus aurantium*	Ethanol, NSAIDs, stress, *H. pylori*, ischemia reperfusion injury and cysteamine-induced ulcers	7.5 mg/kg	Decreased gastric and duodenal lesions, SOD activity, increased gastric mucus production, mucosal MDA levels and GPx and GR activity	[162]
Spathulenol (22.5%), 1,8-cineole (18.3%)	*Croton rhamnifolioides*	Gastric ulcer induced by administration of absolute ethanol, acidified ethanol or indomethacin	100 mg/kg	Modulation of opioid receptors and NO	[163]
**Phenolics and Favonoids**					
Anthocyanins	*Rubus coreanus*	Naproxen-induced gastric ulcer	20, 50 and 80 mg/kg	Via association with regulation of matrix metalloproteinase-2 activity; prevented lipid peroxidation and increased CAT, SOD and GPx activities	[164]
Ellagic and gallic acids	*Euphorbia umbellata* (Pax) Bruyns	Ethanol-induced acute gastric lesions in rats	50, 100, 200 mg/kg	Potent antioxidant activity (PG, NO/cyclic guanosine monophosphate pathway related to antiulcer action)	[165]
Gallic acid	Widespread in plant kingdom	Ethanol-induced gastric ulcerogenesis	25–50 mg/kg	Inhibited gastric acid secretion or through antioxidant action	[166]
Gallic acid and catechin	Widespread in plant kingdom	*In vitro* antimicrobial test on two *H. pylori* strains	1 mg/mL	High inhibitory effect against *H. pylori*	[167]
Gallic acid + famotidine		Aspirin plus pyloric ligation induced ulcer in rat	50 + 10 mg/kg (respectively)	Increased SOD, CAT, GR, GSH and G6PD levels and decreased myeloperoxidase and lipid peroxidation in stomach tissue	[168]
Gallic, chlorogenic and caffeic acids, rutin and quercetin	*Caryocar coriaceum*	Gastric ulcer induced by ethanol, acidified ethanol, acetic acid or indomethacin	100 mg/kg	Opioid receptors, α_2_-adrenergic receptors and primary afferent neurons sensitive to capsaicin	[169]
Garcinol	*Garcinia indica*	Acute ulceration in rats induced by indomethacin and water immersion stress	200 mg/kg	Suppressed superoxide anion, hydroxyl and methyl radicals	[170]
Hesperidin	*Citrus sinensis*	Indomethacin plus hypothermic restrain stress-induced ulceration in rats	150, 300 and 450 mg/kg	Increased GSH and mucin levels, prevented oxidative cell injury	[171]
Quercetin, rutin and kaempferol	Widespread in plant kingdom	gastric damage produced by acidified ethanol in rats	25–100 mg/kg	Inhibited mucosal content of platelet-activating factor	[172]
Rutin and quercetin	*Piper umbellatum* L.	Experimental rodent models	30, 100 and 300 mg/kg	Antioxidant, anti-secretory, anti-inflammatory and mucosa regeneration	[173]
*p*-Coumaric acid	*Macrotyloma uniflorum*	Indomethacin and ethanol-induced ulcer in rats	250 mg/kg	Underlying antioxidant activity (attenuated ulcer elevated MDA levels and reduced GSH, SOD, CAT, GPx and GR levels)	[174]
**Tannins**					
Ellagitannin-rich fraction	*Eucalyptus citriodora*	Ethanol-induced gastric ulceration in rats	25, 50 and 100 mg/kg	Increased GSH and SOD levels	[175]
Tannins (TF) and flavonoids (FF) fractions	*Mouriri pusa*	Gastric ulcer induced by ethanol and acetic acid	25 mg/kg (TF) or 50 mg/kg (FF)	Cell proliferation improved, anti-inflammatory activity by reducing COX-2 levels, increased mucus secretion and angiogenesis	[176]
**Fatty Acids**					
Oleic acid (C18:1 *cis* 9)	Olive oil	Ulcer created in mice using ischemia-reperfusion-induced skin injury	1500 mg/kg	Accelerated ROS and NO synthesis and reduced oxidative damage, inflammatory cells infiltration and TNF-α expression	[177]

CAT, catalase; FF, flavonoids fraction; G6PD, glucose-6-phosphate dehydrogenase; GSH, glutathione; GPx, glutathione peroxidase; GST, glutathione-*S*-transferase; GR, glutathione reductase; HTA, hydroxytormentic acid; HCl, hydrochloric acid; IL-6, interleukin-6; LPO, lipid peroxide; MDA, malondialdehyde; MIC, minimal inhibitory concentration; NO, nitric oxide; PG, prostaglandins; PGE2, prostaglandin E2; ROS, reactive oxygen species; SOD, superoxide dismutase; TF, tannins fraction; TNF-α, tumor necrosis factor-α.
